# Website Use and Associations With Behavior Change and Weight Loss in Cancer Survivors and Their Partners: Secondary Analysis of a Randomized Controlled Trial

**DOI:** 10.2196/86908

**Published:** 2026-01-30

**Authors:** Harleen Kaur, Dori Pekmezi, Tracy E Crane, David Farrell, Laura Q Rogers, Wendy Demark-Wahnefried

**Affiliations:** 1 Department of Psychology and Medicine, Division of Medical Oncology Miller School of Medicine University of Miami Miami, FL United States; 2 Department of Health Behavior School of Public Health University of Alabama at Birmingham Birmingham, AL United States; 3 Department of Medicine, Division of Medical Oncology Miller School of Medicine University of Miami Miami, FL United States; 4 People Designs (United States) Durham, NC United States; 5 Department of Medicine, Division of General Internal Medicine and Population Science School of Medicine University of Alabama at Birmingham Birmingham, AL United States; 6 Department of Nutrition Sciences School of Health Professions University of Alabama at Birmingham Birmingham, AL United States

**Keywords:** cancer survivors, diet, digital health, dyads, partners, physical activity, website

## Abstract

**Background:**

Web-based lifestyle interventions to promote healthy diet and physical activity among cancer survivors and their partners are recent developments; therefore, few studies have reported patterns of website use or associations with behavior change.

**Objective:**

The primary aim was to describe website use in the DUET (Daughters, Dudes, Mothers, and Others Together) trial and examine the associations between website use and changes in diet quality, moderate to vigorous physical activity (MVPA), and body weight.

**Methods:**

This secondary analysis used data from 28 survivor-partner dyads (BMI ≥25 kg/m^2^) randomized to the 6-month DUET web-based weight loss intervention, which released weekly e-learning sessions on diet and exercise. Website use was quantified as weeks of access, time spent, and frequency of page views. Diet quality was assessed using 2-day dietary recalls; MVPA was measured by the Godin Leisure-Time Exercise Questionnaire and accelerometry. Weight was measured on a scale. Website use was summarized descriptively, and associations were examined using Spearman partial correlations.

**Results:**

Participants had a mean age of 58 (SD 12.5) years; 78.6% (44/56) identified as female, 66.1% (37/56) were non-Hispanic White, and 86% (24/28) were breast cancer survivors. On average, participants viewed 11.2 (SD 7.4) weeks of the 24-week intervention, or a total of 312.9 (SD 255.7) minutes per participant. *Sessions* (n=2736), *Home Page* (n=975), and *Tools* (n=967) features showed the highest activity (5885 total page views). Website use was higher among adults aged 65 years and older than younger participants, showcased by duration of use (mean 14.4, SD 7.4 weeks vs mean 9.2, SD 6.8 weeks; *P*=.009), time spent per week (mean 17.0, SD 9.7 minutes vs mean 10.5, SD 10.6 minutes; *P*=.01), and total number of page views (mean 135.7, SD 90 vs mean 85.3, SD 111.9; *P*=.008); higher website use was also reported among women versus men in terms of duration of use (mean 12.8, SD 7.1 weeks vs mean 5.6, SD 5.9 weeks; *P*=.003), time spent per week (mean 14.6, SD 10.3 minutes vs mean 7.4, SD 10.3 minutes; *P*=.02), and total number of page views (mean 120, SD 110.2 vs mean 50.3, SD 64.4; *P*=.01). Diet quality was positively associated with website use (weeks: *r*=0.50; *P*<.001; time: *r*=0.45; *P*<.001; total page views: *r*=0.46; *P*<.001; and sessions page views: *r*=0.39; *P*=.005). Self-reported MVPA was also positively associated with website use (weeks *r*=0.37; *P*=.007; time: *r*=0.36; *P*=.009; total page views: *r*=0.36; *P*=.01; and sessions page views: *r*=0.35; *P*=.01). No significant associations were detected for accelerometry-measured MVPA or weight.

**Conclusions:**

Cancer survivors and their partners engaged with the DUET web-based platform to support diet and physical activity (with use particularly high among older adults and females). However, larger, more diverse dyadic web-based lifestyle interventions are needed to confirm these findings.

**Trial Registration:**

ClinicalTrials.gov NCT04132219; https://clinicaltrials.gov/study/NCT04132219

## Introduction

### Background

The number of cancer survivors in the United States is increasing, with over 18 million currently living with a history of cancer diagnosis [1,2]. While cure rates are encouraging, cancer survivors represent a population with a high burden of comorbidity, including cardiovascular disease, diabetes, and obesity [3,4]. Furthermore, most cancer survivors do not meet the nutrition and physical activity guidelines recommended by the World Cancer Research Fund/American Institute for Cancer Research (WCRF/AICR) and the American Cancer Society (ACS), often reporting low intake of vegetables and fruits (V&Fs) and insufficient physical activity [5-8]. These poor lifestyle practices can further exacerbate existing comorbidities, compounding their impact on long-term survivorship. Despite these challenges, cancer survivors frequently express a strong interest in improving their lifestyle behaviors [9]. Thus, lifestyle interventions that target diet, physical activity, and weight management have been implemented to promote healthier behaviors, support weight loss, and enhance the quality of life of cancer survivors [10-13].

Over the past decade, the landscape of lifestyle interventions for cancer survivors has evolved, with an increasing shift toward accessible and scalable digital delivery methods [14-16]. Among these, web-based lifestyle interventions have emerged as a promising approach to disseminate diet and physical activity guidance to cancer survivors [14,15]. Websites offer several advantages, such as scalability, personalization, cost-effectiveness, and the flexibility for participants to engage with content at their own pace [14,15,17]. To harness these advantages, the SurvivorSHINE pilot study, a 3-week, single-arm web-based lifestyle intervention, was implemented through an interactive website to promote diet and physical activity guidelines among 41 cancer survivors [18,19]. The website incorporated common strategies such as evidence-based diet and exercise knowledge, tools to facilitate behavior change, and resources for self-monitoring and goal setting [18]. The study reported that cancer survivors perceived the SurvivorSHINE website as a user-friendly platform that provided trustworthy information on diet and exercise, and reported improvements in knowledge related to diet and exercise [18].

Building upon the frameworks and strategies of the SurvivorSHINE intervention, a more refined website was developed that included 24 weekly serialized, interactive sessions, as well as additional tools to support behavior change not only among cancer survivors interested in cancer control, but also among their family members and friends for the purposes of cancer prevention. This newly developed website was then evaluated for feasibility and its impact on various health outcomes in a randomized controlled trial, known as DUET (Daughters, Dudes, Mothers, and Others Together; NCT04132219) that targeted cancer survivors and their chosen partners [20,21].

The DUET trial was 6 months in duration and evaluated the web-based weight loss intervention against a waitlist control among 112 participants (56 dyads, each consisting of a cancer survivor and a chosen partner) [20,21]. Results showed that dyads in the intervention arm achieved significant weight loss, along with improvements in diet quality and physical activity levels compared to the control arm [21]. A mediation analysis further revealed that reductions in perceived dietary barriers significantly contributed to the observed weight loss, highlighting the effectiveness of the intervention’s strategies, which were based on social cognitive theory [22-24]. These findings underscore the potential of minimal-touch, web-based lifestyle interventions to facilitate meaningful behavior change and weight loss among cancer survivors and their partners. However, to optimize delivery and behavioral outcomes, it is important to understand how cancer survivors and their partners used the DUET website. While many web-based interventions focus on evaluating behavioral outcomes, relatively few have described website use or examined the association between website use and changes in diet, physical activity, and weight loss. Exploring patterns of use within the DUET website may provide insight into participant interaction with web-based platforms and inform refinements for future web-based lifestyle interventions for cancer survivors and their partners.

### Aim and Objective

This secondary analysis aims to describe website use in the DUET trial, and also examine the associations between website use (as measured by number of weeks of website use, average time spent, total page views, and session page views) and changes in diet quality, moderate to vigorous physical activity (MVPA), and weight.

## Methods

### Study Design

The DUET study was a 2-arm single-blinded randomized controlled trial that enrolled 112 cancer survivors and their chosen partners (56 dyads). Dyads were randomized to either the 6-month web-based weight loss intervention or a waitlist control arm. This secondary analysis focuses on the subset of 56 participants (28 dyads) assigned to the DUET intervention arm, as website usability data were only collected for this group. Full details of the trial methods, primary outcomes, and mediation analysis have been published elsewhere [20-22].

### Study Participants

Cancer survivors were identified through multiple recruitment strategies, including cancer registries, self-referrals, and curated lists of individuals who had previously expressed interest in lifestyle interventions. Recruitment efforts focused on cancer survivors who had completed treatment for obesity-related cancers with a 5-year survival rate of ≥70% (eg, localized renal, locoregional ovarian, colorectal, prostate, endometrial, or female breast cancer). Potential cancer survivors were contacted by mail with telephone follow-up, and, if interested, were screened by study staff for the following inclusion criteria: (1) BMI ≥25 kg/m²; (2) V&F intake <2.5 cups/day; (3) engaging in <150 minutes/week of MVPA; and (4) routine access to the internet via a computer, tablet, or mobile phone. Eligible cancer survivors were asked to identify a partner who lived nearby (within 10 minutes by car) and interacted with them at least biweekly; partners were screened using the same criteria, excluding a cancer diagnosis. However, partners with a history of cancer were eligible.

### Study Protocol

Eligible dyads were provided with an overview of the study protocol, and written informed consent was obtained electronically using Adobe Sign [25]. After completion of baseline assessments, dyads were randomized to either the 6-month web-based weight loss intervention or a waitlist control arm. Those assigned to the intervention arm were granted access to the DUET website via an electronic link and instructed to create a secure profile using a username and password unique for each survivor and partner [26]. Dyads were encouraged to log in weekly via text messages and engage with the key features of the DUET website throughout the 6-month intervention period. Follow-up assessments were conducted at 6 months, after which waitlist control dyads were provided access to the DUET intervention. Additional details on the study procedures have been published previously [20].

### DUET Intervention Website

The DUET intervention was theoretically grounded in social cognitive theory and incorporated elements from interdependence theory and the theory of communal coping to support both individual and dyadic behavior change by targeting diet and exercise barriers, enhancing social support, and building self-efficacy [23,24,27,28]. DUET was adapted from 2 prior evidence-based lifestyle interventions, Daughters and Mothers Against Breast Cancer and SurvivorSHINE, and delivered via a secure, interactive website that served as the primary web-based platform for intervention delivery [18,26,29]. Details on the intervention development have been published previously [20].

The DUET website included a total of 9 key features: *Home Page, My Profile, Sessions, Healthy Weight, Healthy Eating, Exercise, Tools, News You Can Use, and Team Support.* Upon account creation, participants accessed the *My Profile* feature to enter demographic and lifestyle data, including their current weight, height, and responses to 1-item questions that assessed their dietary intake (eg, consumption of V&Fs, whole grains, red and processed meats, added sugars, and alcohol), and frequency of snacking and physical activity (both endurance and resistance exercise). Cancer survivors also provided details on diagnosis and treatment, which informed tailored feedback on overcoming treatment-related challenges and generating dietary, physical activity, and weight management goals, as described previously [20-22]. The *Home Page* displayed a “Tip of the Day” designed to encourage ongoing engagement with the website, along with visual indicators showing participants’ completed weekly sessions and the next upcoming session. It also included direct links to other key features of the website (ie, *Healthy Weight, Healthy Eating, Exercise, Tools, News You Can Use, and Team Support)*.

The *Sessions* feature included 24 weekly interactive e-learning modules (~15 minutes each), designed using Articulate Storyline software [30]. Sessions were released sequentially each week over the 24 weeks and introduced via a Monday SMS “push” text message, with additional text messages sent on Wednesdays and Fridays to reinforce continued engagement. These interactive e-learning sessions were designed to provide information on the WCRF/AICR and ACS diet and physical activity guidelines and equip participants with practical and actionable strategies to support behavior change and weight management [5,6]. Dietary recommendations were supported by sessions focused on promoting the consumption of V&Fs, whole grains, and legumes, and limiting red and processed meat, added sugar, alcohol, and snacking, with additional sessions focused on portion control, grocery shopping, and food preparation to support healthy eating habits. Physical activity recommendations were supported by sessions focused on aerobic, resistance, balance, and flexibility exercises, with emphasis on goal setting and problem solving to help participants gradually achieve the goal of 150 minutes of MVPA per week. Details on weekly topics have been published previously [20].

The *Healthy Weight* feature was designed to support self-monitoring of weight by providing an interactive bar graph that tracked participants’ current weight and healthy weight. Accompanying educational materials helped contextualize these values by explaining the concept of a healthy weight and its relevance to cancer prevention and survivorship. The website delivered tailored guidance to help participants progress toward a healthy weight by supporting caloric restriction and strategies to promote gradual weight loss of approximately 0.5 kg/week [31].

The *Healthy Eating* feature supported self-monitoring of dietary goals aligned with the WCRF/AICR and ACS guidelines. Participants could record their current intake of key dietary components, that is, V&Fs (≥5 servings/day), whole grains (≥50% of total grain intake), added sugars (≤6 teaspoons/day), avoid snacks, red and processed meats (≤18 ounces/week), and alcohol (≤1 drink/day), through an interactive bar graph that visually displayed their reported intake alongside goal targets. To further support dietary changes, the feature provided educational resources on each dietary component.

The *Exercise* feature supported self-monitoring of physical activity through tailored recommendations based on participants’ self-reported activity levels. Physical activity goals were aligned with WCRF/AICR and ACS guidelines, encouraging participants to achieve at least 150 minutes of MVPA and engage in strength training 2-3 times per week.

The *Tools* feature provided a centralized hub for participants to access downloadable materials that supported both dietary and physical activity behaviors aimed at achieving a healthy weight. This feature included 11 distinct resources: (1) BMI calculator, (2) calorie calculator, (3) sample meal plans, (4) food exchange lists, (5) SMART goal templates, (6) serving size guides, (7) fast food guide, (8) grocery lists and shopping tips, (9) calorie-burning guide, (10) exercise logs, and (11) tools for tracking Fitbit data (note: all DUET participants regardless of randomization status, received a Fitbit Inspire and were encouraged to use it during the study period; however, Fitbit data were not integrated into the DUET website) [32]. These resources were designed to offer participants additional support, practical strategies, and accessible tools to enhance self-efficacy throughout the intervention.

The *News You Can Use* feature provided brief, evidence-based summaries on current research related to cancer survivorship, diet, and exercise. The *Team Support* section provided strategies to strengthen dyadic communication, foster mutual goal setting, and enhance social support; it also allowed participants to directly connect with study staff for additional guidance and support.

### Measures

#### Demographics

Cancer type and time since diagnosis were obtained from cancer registries or verified by treating physicians for self-referred participants. Demographic information, including age, sex, race, residence, educational status, employment, and income, was self-reported via electronic surveys completed at baseline. Cohabitation was assessed by comparing mailing addresses; dyads with the same address (0 miles) were classified as cohabitating, and those with different addresses (>0 miles) as noncohabitating.

#### Diet Quality, MVPA, and Weight

Dietary intake was assessed at baseline and 6 months via two 24-hour dietary recalls (1 weekday and 1 weekend day) conducted by a registered dietitian over the telephone. The Automated Self-Administered 24-hour Dietary Assessment Tool was used to capture dietary intake data. Diet quality was evaluated using the Healthy Eating Index 2015 [33,34].

MVPA was assessed both objectively and subjectively at baseline and 6 months. Participants wore ActiGraph accelerometers for 7 consecutive days. Data were then processed using ActiLife software following standardized procedures to calculate average weekly minutes of MVPA [35,36]. Self-reported MVPA was captured using the Godin Leisure-Time Exercise Questionnaire, a validated tool frequently used in cancer survivorship research [37].

Weight was measured remotely at baseline and 6 months. Each survivor-partner dyad completed the virtual assessment together via Zoom with study staff [38]. Participants used a digital bathroom scale to report their weight; a scale was provided with the assessment materials for those who did not own one. During the virtual assessment, trained staff instructed participants to wear light clothing and remove their shoes, and partners assisted in holding the camera and angling it so that study staff could verify the weight displayed on the digital scale. Additional details on the remote assessment protocol and its validity have been described previously [39].

#### Website Use Metrics

Website use was assessed using tracking data logged by the DUET website platform. Each participant was assigned a unique website username and password, which was linked to their website ID and further connected to their study ID, allowing for the tracking of individual-level website activity over the 24-week intervention period. Time-stamped data recorded the days, times, and pages accessed by participants (eg, *Home Page, My Profile, Sessions, Healthy Weight, Healthy Eating, Exercise, Tools, News You Can Use, and Team Support*). From these data, key website use metrics were derived, including (1) the total number of weeks participants accessed the website, defined as the number of distinct weeks during the 24-week intervention period in which any website activity was recorded; (2) the average time spent on the website per day of use, calculated using time-stamped activity logs that captured first and last activity on a given day; (3) the total number of page views, defined as the cumulative number of times participants navigated to individual pages within each of the website’s key features; and (4) the total number of session page views, defined as the number of times participants accessed the e-learning *Sessions* feature.

### Statistical Analysis

Website usability was analyzed using descriptive statistics, including means, SDs, and ranges for continuous variables (eg, number of weeks accessed, time spent on the website, and page views). Website usability metrics were described for the total sample and stratified by dyad member (cancer survivors or partners), clinical (ie, cancer type and time since diagnosis) and sociodemographic factors (ie, age, sex, race, residence, educational status, employment, and income) and cohabitation status (cohabitate or did not cohabitate), with all stratification variables dichotomized for analysis. To examine differences in website use between dyad members, clinical and sociodemographic factors, and cohabitation status, assumptions of normality were assessed using Shapiro-Wilk tests and visual inspection of histograms and Q-Q plots. Given that usability metrics were not normally distributed, Wilcoxon rank-sum tests were conducted to compare median differences in website use between dyad members, clinical and sociodemographic factors, and cohabitation status. Assumptions of normality and linearity were assessed for the independent (website use metrics) and dependent variables (diet quality, MVPA, and weight). Given evidence of nonnormal distributions, likely influenced by the modest sample size and potential nonlinear relationships, nonparametric methods, such as bivariate Spearman partial rank correlation analyses, were conducted to examine associations between website use and diet quality, MVPA, and weight. Correlation coefficients were generated using 6-month outcome values as dependent variables, adjusting for baseline values of the respective outcome, as well as age, sex, and race to account for potential confounding and assess change over time. Given this was a secondary analysis and not prospectively powered for these aims, we conducted a post hoc power calculation to aid interpretation of the correlation analyses. With a total sample size of 56 participants, the study had ≥80% power to detect correlations of approximately *r*≥0.40. Missing data were handled using complete-case analysis. One participant had missing diet quality data at 6-month follow-up, and 13 participants had missing accelerometer-measured MVPA data at baseline (n=8) and 6-month follow-up (n=5); these cases were excluded from their respective models. No adjustments for multiple comparisons were made, given the exploratory nature of this secondary analysis. However, to address the increased type I error risk from multiple correlations, 95% CIs were reported with *P* values to assist in interpreting the precision of the coefficients. All analyses were conducted using SAS (version 9.4, SAS Institute Inc), and statistical significance was set at *P*<.05 [40].

### Ethical Considerations

The DUET study was approved by the Institutional Review Board at the University of Alabama at Birmingham (IRB# 300003882) and was registered with ClinicalTrials.gov (NCT04132219). All participants provided written informed consent, and study procedures were conducted in accordance with the ethical standards of the Declaration of Helsinki to maintain participant confidentiality. No compensation was provided to study participants.

## Results

### Sample Characteristics

The average age of the sample was 58 (SD 12.5) years, with survivors averaging 60 (SD 11.2) years and partners 56 (SD 13.7) years. The majority of cancer survivors (24/28, 85.7%) had a breast cancer diagnosis, with an average time since diagnosis of approximately 71 (SD 80.4) months (6 years). Most participants identified as female (44/56, 78.6%), non-Hispanic White (37/56, 66.1%), and residents of urban areas (53/56, 94.6%). Employment status was evenly split between employed (30/56, 53.6%) and retired (26/56, 46.4%), and most (45/56, 80.4%) reported an annual household income above US $50,000 per year (Table 1).

**Table 1:** Characteristics of 56 cancer survivors and their chosen partners randomized to the intervention arm, stratified by dyad status.

Characteristics		Total sample		Survivor		Partner			*P* value^a^	
Age (years), mean (SD; range)		58.1 (12.5; 23-78)		60.0 (11.2; 32-78)		56.3 (13.7; 23-74)			.28	
Months from diagnosis, mean (SD; range)		71 (80.4; 10-303)		71 (80.4; 10-303)		—^b^			—^b^	
BMI (kg/m^2^), mean (SD; range)		31.4 (4.9; 25-45)		32.0 (5.4; 25-44)		30.9 (4.4; 25-45)			.41	
Diet quality (HEI^c^), mean (SD; range)		53.1 (12.8; 29-87)		53.9 (13.7; 30-87)		52.2 (12; 29-81)			.62	
MVPA^d^ (min/week), mean (SD; range)		43.8 (60.5; 0-280)		48.5 (67.8; 0-280)		39.1 (52.9; 0-225)			.57	
**Cancer type^e^, n (%)**							<.001			
	Breast	25 (44.6)	24 (85.7)		1 (3.6)					
	Other^f^	7 (12.5)	4 (14.3)		3 (10.7)					
**Sex, n (%)**										.05
	Male	12 (21.4)	3 (10.7)		9 (32.1)					
	Female	44 (78.6)	25 (89.3)		19 (67.9)					
**Race, n (%)**										.78
	Non-Hispanic White	37 (66.1)	19 (67.9)		18 (64.3)					
	Non-Hispanic Black or other^g^	19 (33.9)	9 (32.1)		10 (35.7)					
**Residence, n (%)**										.55
	Urban	53 (94.6)	27 (96.4)		26 (92.9)					
	Rural	3 (5.4)	1 (3.6)		2 (7.1)					
**Educational status, n (%)**										.13
	High school or less	8 (14.3)	2 (7.1)		6 (21.4)					
	Some college or more	48 (85.7)	26 (92.9)		22 (78.6)					
**Employment, n (%)**										>.99
	Employed	30 (53.6)	15 (53.6)		15 (53.6)					
	Retired or other^h^	26 (46.4)	13 (46.4)		13 (46.4)					
**Income, n (%)**										.09
	Less than US $50,000/year	11 (19.6)	8 (28.6)		3 (10.7)					
	More than US $50,000/year	45 (80.4)	20 (71.4)		25 (89.3)					

^a^*P* values were calculated using independent samples *t* tests for continuous variables (based on equal or unequal variances as appropriate) and chi-square tests for categorical variables, comparing survivors vs partners, significance set at *P*<.05.

^b^Data on months since diagnosis is unavailable for partners.

^c^HEI: Healthy Eating Index 2015.

^d^MVPA: moderate to vigorous physical activity.

^e^Total percentage does not sum to 100% due to the inclusion of nonsurvivors who were not diagnosed with cancer.

^f^Other cancer diagnoses include prostate, colorectal, gynecologic, and renal.

^g^Other race includes Hispanic ethnicity, accounting for 1%.

^h^Other employment includes student and disabled.

### DUET Website Use

On average, participants accessed the DUET website for 11.2 (SD 7.4; range 0-24) weeks and spent a total of 312.9 (SD 255.7; range 0-1119) minutes on the platform, or about 13 (SD 10.7; range 0-46.6) minutes per week over the 24-week intervention period. A total of 5885 page views were recorded, with an average of 105.1 (SD 105.7; range 0-545) page views per participant. Cancer survivors used the website more frequently than their partners, accessing it for a longer duration (mean 13.0, SD 7.2 weeks vs mean 9.5, SD 7.4 weeks; *P*=.08), spending more time per week (mean 15.6, SD 11.4 minutes vs mean 10.5, SD 9.4 minutes; *P*=.07), and recording a higher average number of page views (mean 124.2, SD 114.2 vs mean 86.0, SD 94.5; *P*=.07); however, these differences were not statistically significant (Table 2).

**Table 2:** Daughters, Dudes, Mothers, and Others Together website usability over the 24-week intervention period for the total sample (n=56) and stratified by dyad status.

Engagement metrics	Total sample, mean (SD; range)	Survivor, mean (SD; range)	Partner, mean (SD; range)	*P* value^a^
Weeks participants accessed website	11.2 (7.4; 0-24)	13.0 (7.2; 0-24)	9.5 (7.4; 0-23)	.08
Total time spent on website (min)	312.9 (255.7; 0-1119)	373.8 (273.3; 0-1119)	252.0 (225.5; 0-670)	.07
Time spent on website per week (min)	13.0 (10.7; 0-46.6)	15.6 (11.4; 0-46.6)	10.5 (9.4; 0-27.9)	.07
Total page views per user (n=5885)	105.1 (105.7; 0-545)	124.2 (114.2; 0-545)	86.0 (94.5; 0-352)	.07

^a^*P* values represent comparisons between survivors and partners using Wilcoxon rank-sum tests due to the nonnormal distribution of website usability metrics, significance set at *P*<.05.

Website use differed significantly by age, time since diagnosis, and sex. Older adults aged ≥65 years demonstrated higher website use compared to younger participants (<65 years), accessing it for a longer duration (mean 14.4, SD 7.4 weeks vs 9.2, SD 6.8 weeks; *P*=.009), spending more time per week (mean 17.0, SD 9.7 minutes vs mean 10.5, SD 10.6 minutes; *P*=.01), and recording a higher average number of total page views (mean 135.7, SD 90 vs mean 85.3, SD 111.9; *P*=.008). Survivors who were 5 or more years post diagnosis also accessed the website for longer duration than those more recently diagnosed (<5 years; mean 18.4, SD 6.3 weeks vs mean 10.8, SD 6.4 weeks; *P*=.009). Additionally, females used the website significantly more than males, accessing it for a longer duration (mean 12.8, SD 7.1 weeks vs mean 5.6, SD 5.9 weeks; *P*=.003), spending more time per week (mean 14.6, SD 10.3 minutes vs mean 7.4, SD 10.3 minutes; *P*=.02), and recording a higher average number of total page views (mean 120, SD 110.2 vs mean 50.3, SD 64.4; *P*=.01; Table 3).

**Table 3:** Website use stratified by clinical characteristics, sociodemographic factors, and cohabitation status.

Measures			Weeks							Average time							Total page views							Session page views						
			Mean (SD)		Test statistic^a^		*P* value		Mean (SD)			Test statistic		*P* value		Mean (SD)			Test statistic		*P* value		Mean (SD)			Test statistic		*P* value		
**Age**					6.83		.009					6.55		.01					7.03		.008					6.34		.01		
	<65 years	9.17 (6.8)						10.5 (10.6)							85.3 (111.9)							38.4 (35.2)								
	≥65 years	14.4 (7.4)						17 (9.7)							135.7 (90)							65 (38.5)								
**Time since diagnosis**					6.88		.009					1.62		.20					2.57		.11					0.622		.43		
	<5 years	10.8 (6.4)						14 (11.9)							108.4 (117.8)							52.5 (35.1)								
	≥5 years	18.4 (6.3)						19.4 (9.6)							163.6 (100.9)							65.3 (32.3)								
**Cancer type**					0.64		.42					0.03		.87					0.53		.47					1.15		.28		
	Breast	13.6 (7.2)						15.3 (11.3)							122.9 (119.5)							54.8 (37.1)								
	Other	11.6 (4.9)						15.9 (9.7)							129.6 (81)							27 (36.7)								
**Sex**					8.82		.003					5.70		.02					6.69		.01					6.39		.01		
	Female	12.8 (7.1)						14.6 (10.3)							120 (110.2)							54.8 (37.1)								
	Male	5.6 (5.9)						7.4 (10.3)							50.3 (64.4)							27 (36.7)								
**Race**					0.02		.89					0.08		.78					0.11		.75					0.01		.91		
	Non-Hispanic White	11.4 (6.8)						12.9 (9.6)							100.2 (86.2)							46.4 (33.2)								
	Non-Hispanic Black	10.9 (8.6)						13.3 (12.5)							113.9 (136)							53.4 (47.1)								
**Residence**					0.01		.91					0.41		.52					0.28		.60					0.90		.34		
	Urban	11.2 (7.3)						12.7 (10.3)							104 (106.8)							47.2 (36.3)								
	Rural	12.3 (11)						18.4 (17.1)							123.7 (98.1)							77.7 (70.3)								
**Education**					0.95		.33					0.01		.92					0.08		.78					0.00		.98		
	High school or less	8.9 (6.3)						12 (10.5)							116 (131.7)							45.9 (37.8)								
	Some college or above	11.6 (7.6)						13.2 (10.8)							103.3 (102.2)							49.4 (38.9)								
**Employment**					1.29		.26					0.14		.71					0.35		.55					0.14		.71		
	Employed	10.4 (6.3)						12.9 (11.7)							101.8 (117.1)							47.6 (39.7)								
	Retired or other	12.2 (8.5)						13.3 (9.5)							108.9 (92.8)							50.3 (37.7)								
**Income**					0.08		.78					0.45		.50					0.18		.67					0.81		.37		
	Less than US $50k/year	10.6 (5.9)						15.9 (13.9)							132 (152.8)							59.7 (44.9)								
	More than US $50k/year	11.4 (7.8)						12.3 (9.8)							98.5 (91.7)							46.2 (36.8)								
**Cohabitation**					2.23		.14					1.54		.21					0.40		.53					2.01		.16		
	Cohabitate	9.5 (8.1)						11.1 (10.3)							93.1 (91)							42.2 (39.8)								
	Do not cohabitate	12.5 (6.7)						14.5 (10.8)							114.1 (116)							53.8 (37.2)								

^a^Differences between groups were assessed using the Mann-Whitney *U* test, implemented via the Wilcoxon rank-sum approach. A chi-square approximation was used to derive the test statistics, with statistical significance defined as *P*<.05.

**Figure 1:**
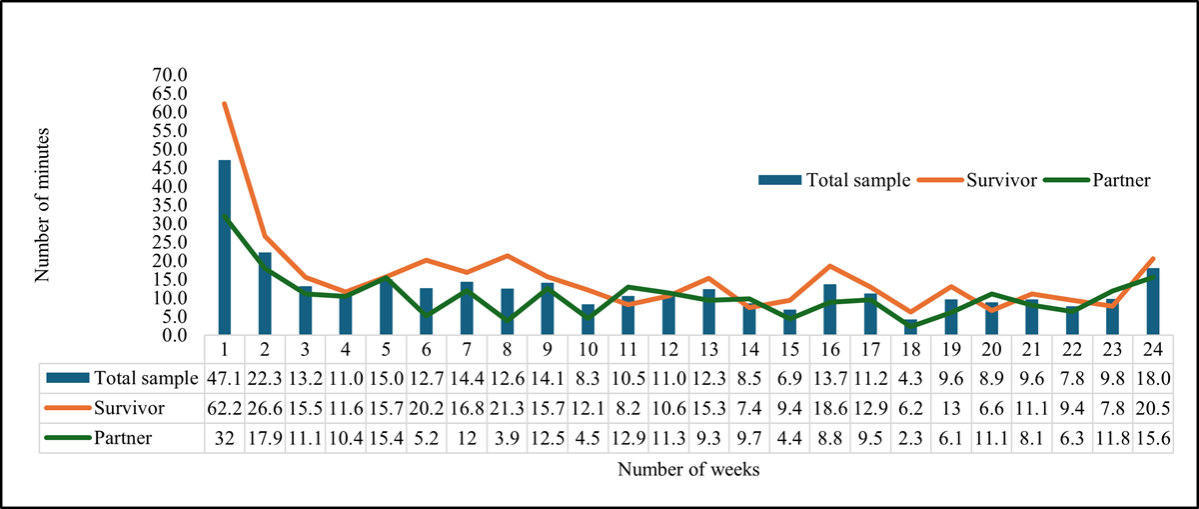
Average weekly time spent on the Daughters, Dudes, Mothers, and Others Together (DUET) website during the 24-week intervention period by the total sample, highlighting high and low engagement weeks stratified by dyad status. All weekly DUET sessions were designed to take 15 minutes or less, except for the initial onboarding session.

Website use was generally highest during the initial 9 weeks of the intervention, although some fluctuations were observed, particularly in weeks 4, 6, and 8. Website use gradually declined over time, with a slight increase observed in the final week of the intervention. Cancer survivors spent more time on the website each week compared to their partners, with notable peaks observed among cancer survivors at weeks 1, 2, 6, 8, 13, 16, 19, and 24 (Figure 1).

**Figure 2:**
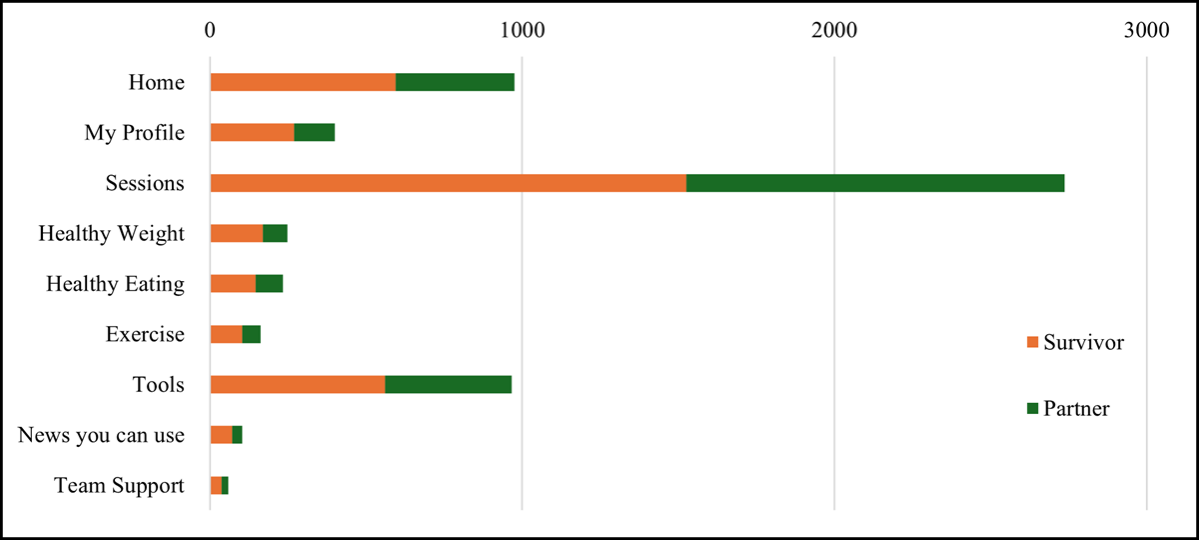
Page view distribution across key features of the Daughters, Dudes, Mothers, and Others Together website stratified by dyad status (n=5885 total page views).

A total of 5885 page views were recorded throughout the intervention period across the 9 key website features. The highest number of page views was observed for the weekly interactive e-learning *Sessions* feature (n=2736), followed by the *Home Page* (n=975) and *Tools* feature (n=967). The remaining features, *My Profile* (n=400), *Healthy Weight* (n=248), *Healthy Eating* (n=234), *Exercise* (n=162), *News You Can Use* (n=104), and *Team Support* (n=59), had comparatively fewer page views (Figure 2).

**Figure 3:**
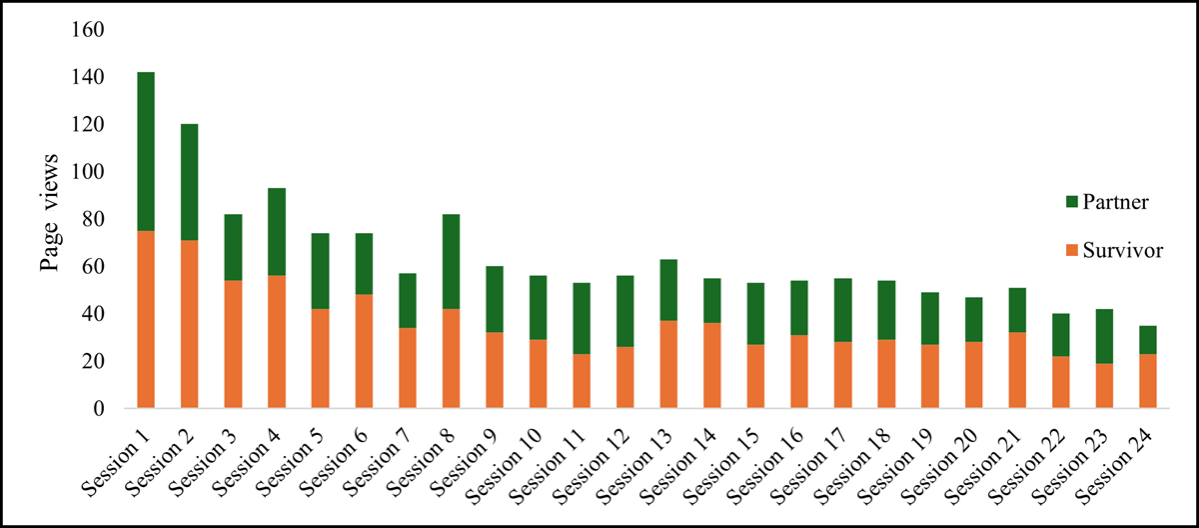
Page view distribution of weekly released diet and exercise sessions accessed by cancer survivors and partners (n=2736 total session page views). Top 3 most viewed sessions for the total sample: Get on Track for Success (session 2), Moving Towards Better Health (session 4), and Been Resisting “Resistance” Exercises (session 8). Bottom 3 least viewed sessions for the total sample: Want to Join the Party Without Blowing Your Diet (session 20), Why Am I Hungry All the Time (session 22), Are Supplements Really Good for You (session 23).

Figure 3 further illustrates the distribution of 2736 page views across the weekly e-learning *Sessions* feature over the 24-week intervention period. Page views for the weekly sessions were highest during the early weeks of the intervention, particularly sessions 1 through 8, and gradually declined over time. The onboarding session received the greatest number of views, and the three most frequently viewed sessions were (1) Get on Track for Success (session 2), (2) Moving Towards Better Health (session 4), and (3) Been Resisting “Resistance” Exercises (session 8). Conversely, the three least viewed sessions were (1) Want to Join the Party Without Blowing Your Diet? (session 20), (2) Why Am I Hungry All the Time? (session 22), (3) Are Supplements Really Good for You? (session 23).

The *Tools* feature accounted for the third highest number of page views (n=967), following the *Home page* and *Sessions*, with participants most frequently viewing tools such as Sample Meal Plans (n=121 page views), Tracking with Fitbit (n=119), Exercise Logs (n=65), BMI Calculator (n=65), Fast Food Menu Maven (n=64), Calorie Calculator (n=62) and Calorie Burning Guide (n=60; Multimedia Appendix 1). The *Healthy Weight, Healthy Eating, and Exercise* features were also accessed throughout the intervention period. Within these features, the most commonly viewed content included “Common Questions About Weight Management” (n=20 page views) under the Healthy Weight category, “Increasing V&F Intake” (n=10 page views) under Healthy Eating, and “Leg Strengthening Exercises” (n=19 page views) under the Exercise category (Multimedia Appendix 2)

### Website Use and Behavioral Associations

Diet quality was positively associated with website use, weeks (*r*=0.50; *P*<.001), time (*r*=0.45; *P*<.001), total page views (*r*=0.46; *P*<.001), and sessions page views (*r*=0.39; *P*=.005). Self-reported MVPA was also positively associated with website use, weeks (*r*=0.37; *P*=.007), time (*r*=0.36; *P*=.009), total page views (*r*=0.36; *P*=.01), and sessions page views (*r*=0.35; *P*=.01). However, no statistically significant associations were detected for accelerometry-measured MVPA or weight (*P*>.05; Table 4).

**Table 4:** Bivariate associations between website usability and Healthy Eating Index 2015 diet quality, moderate to vigorous physical activity (MVPA), and weight at 6-months. Bivariate associations were performed using Spearman partial correlations rank analysis for nonnormally distributed data. Adjusted for age, sex, race, and baseline outcome variables.

Measures	Weeks		Average time		Total page views			Session page views		
	*r* (95% CI)	*P* value	*r* (95% CI)	*P* value	*r* (95% CI)	*P* value	*r* (95% CI)		*P* value	
Diet quality	0.50 (0.25 to 0.68)	<.001	0.45 (0.20 to 0.65)	<.001	0.46 (0.21 to 0.65)	<.001	0.39 (0.12 to 0.60)		.005	
Self-reported MVPA	0.37 (0.10 to 0.58)	.007	0.36 (0.09 to 0.57)	.009	0.36 (0.09 to 0.57)	.01	0.35 (0.08 to 0.57)		.01	
Accelerometer MVPA	0.15 (–0.16 to 0.44)	.33	0.06 (–0.25 to 0.37)	.68	0.03 (–0.28 to 0.34)	.82	0.04 (–0.27 to 0.35)		.79	
Weight	–0.17 (–0.42 to 0.11)	.24	–0.06 (–0.32 to 0.22)	.67	–0.13 (–0.39 to 0.15)	.35	–0.11 (–0.38 to 0.16)		.41	

## Discussion

### Primary Findings

There are very few web-based lifestyle interventions for cancer survivors and their support partners that use evidence-based theoretical constructs to promote healthful diet and physical activity behaviors [14]. As a result, no studies to date have described patterns of website use to inform the design and delivery of future dyadic, web-based lifestyle interventions. This study is among the first to address this gap by providing a detailed analysis of website use patterns among cancer survivors and their chosen partners randomized to the DUET intervention. Our findings suggest that cancer survivors and their partners used the DUET website to learn about healthy lifestyle guidelines, as reflected by website use metrics: on average, participants logged into roughly half of the 24-week content, interacting for a total of 313 minutes with the program, for which the highest page view activity was observed for *Sessions* (n=2736). Older adults and females engaged with the website to a significantly greater degree compared to younger participants and males, as reflected by weekly website activity (frequency, user time, and page views). Moreover, higher levels of website use were significantly associated with improvements in diet quality and self-reported MVPA. However, no significant associations were observed between website use and accelerometer-measured MVPA and weight.

### Comparison With Previous Literature

Due to variability in how website usability metrics are reported, direct comparisons of website use across studies are challenging. However, findings from 3 pilot web-based lifestyle interventions implemented among cancer survivors generally align with our data that cancer survivors engage with a lifestyle website approximately once per week, particularly during the initial phase of the study. For example, the SurvivorSHINE study reported an average of 1.5 log ins per week over a 2-week period [41]. Similarly, in the A Lifestyle Intervention Via Email study, breast cancer survivors visited the website for an average of 9.6 out of 12 weeks in the physical activity arm and 10.7 out of 12 weeks in the diet arm [42]. A study by Blarigan and colleagues [43] reported that colorectal cancer survivors in the intervention arm accessed the website on a median of 13 out of 84 days. Despite the 24-week DUET intervention being roughly twice as long as web-based interventions used in previous studies, we observed comparable weekly website use during the initial 12 weeks of the program, suggesting sustained and moderate website use among dyads during the initial phases of the intervention. Our findings also showed that dyads spent a total of 313 minutes on the website, which differs from the SurvivorSHINE study (94 minutes) but aligns closely with the Breast Cancer eHealth Self-Management study (337 minutes) [41,44]. The difference in website use observed in our study compared to SurvivorSHINE is likely due to the longer intervention duration and the inclusion of weekly serialized e-learning sessions and videos, which were not part of the SurvivorSHINE’s 2-week intervention.

This study also examined page view activity to assess participant usability with various DUET website features. The most frequently accessed feature was the interactive e-learning “*Sessions*,” followed by the “*Home Page*” feature. Prior systematic reviews have emphasized the importance of interactive educational modules to promote real-time engagement with behavior change content [45-47]. The DUET “*Sessions*” were developed with this framework in mind, incorporating brief, skill-building activities designed to help both survivors and their support partners apply evidence-based knowledge to everyday challenges. These sessions encouraged teamwork and joint problem-solving to support healthier behaviors, a strategy supported by the theory of communal coping and prior studies [27,48,49]. Participants also received 3 weekly text messages reminding them to visit the website and engage with the weekly content, which likely contributed to the high usability observed with the “*Sessions*” feature. The brief, focused, and interactive nature of the sessions may have further enhanced their appeal. Similar to findings from the SurvivorSHINE study, the “*Home Page*” was also commonly viewed, likely because it automatically loaded each time participants accessed the website, allowing them to engage with core behavior change constructs such as self-monitoring, goal setting, and motivation [41].

Consistent with prior research conducted in samples without a history of cancer, our findings indicate that website use was significantly higher among older adults (aged ≥65 years) and female participants. For example, Graham et al [50] reported that older adults using the commercially available Lark Health digital platform logged more meals (174 vs 89) and used more self-monitoring devices (39 vs 28) compared to younger adults. Relatedly, a scoping review found that among adults aged 50 years and older, structured, tailored digital programs were well-accepted [51]. Similarly, a growing body of literature has consistently shown that women are more likely to seek health-related information, engage with online health platforms, and participate in digital lifestyle interventions compared to men [14,52,53]. In our study, this trend may be amplified by the DUET program’s structured, self-paced design and tailored content, which likely resonated with older and female participants who may prefer individualized guidance and flexibility with web-based programs [52,54].

Bivariate analyses from this study showed that website use was significantly associated with improvements in diet quality and self-reported MVPA at 6 months. The evidence on whether website use improves diet quality remains unclear, largely due to heterogeneity in how diet quality is measured across studies. For instance, our findings differ from those of a systematic review and meta-analysis of 29 studies in adults with chronic conditions, which found no evidence for website use to improve overall diet quality [55]. However, most studies in this review were short in duration (<3 months) and relied on self-reported measures; in contrast, our study used interviewer-administered 24-hour dietary recalls, the gold standard for diet quality assessment, which may be more sensitive to detecting change. Our findings are more consistent with systematic reviews and several studies that have reported moderate to strong associations between website use and increased physical activity using self-reported measures [41,55-57]. One possible reason the DUET website led to improvements in diet quality and self-reported MVPA is its inclusion of a range of relevant content for cancer survivors and their partners, such as guidance on V&F intake, reducing processed foods, portion control, setting SMART goals, and providing tools for tracking and self-monitoring diet and physical activity behaviors. However, it is important to note that diet quality and self-reported MVPA models were modestly significant, and may not withstand correction for multiple testing and should be interpreted as hypothesis-generating.

Despite these positive associations, our analysis did not find a significant association between website use and accelerometer-measured MVPA and weight. Our findings for not detecting significant associations with subjectively measured physical activity aligns with findings from previous studies [55,56], but, they contrast with findings from the Commonwealth Scientific and Industrial Research Organisation (CSIRO) Total Wellbeing Diet Online program and the Weight Loss Maintenance (WLM) trial, which found that website use was associated with greater weight loss and reduced weight regain, respectively [58,59]. However, the CSIRO program was commercially delivered and relied on participant self-reported weights within the platform, while the WLM trial focused on weight maintenance rather than weight loss [58]. Several methodological, individual, and behavioral level considerations may help explain the lack of association between website use and accelerometer-measured MVPA and weight [60]. Our power calculation confirmed that with 56 participants, the study was powered only to detect correlations of approximately *r*≥0.40. Given that the association between website use and accelerometer-measured MVPA and weight observed in our data was substantially smaller (*r*<0.20), the study was likely underpowered to detect this relationship. Additionally, differences in how outcomes were measured may further clarify the findings. Website use, diet quality, and self-reported MVPA rely on participant interaction and reporting, which may naturally relate to one another and explain shared method variance. In contrast, accelerometer-measured MVPA and weight are objective measures that do not rely on participant reporting, and therefore their associations with website use may be smaller and more difficult to detect. Beyond methodological explanations, individual and behavioral factors may also play a role. For instance, website use may support behavior change but not be sufficient on its own to produce weight loss, as some participants may have relied less on the website once new habits were established. Additionally, weight loss may be influenced more by theoretical and behavioral mechanisms (ie, reduced perceived barriers, self-monitoring, calorie restriction, increased accountability from participating in the study, or lifestyle changes occurring outside the platform). These possibilities suggest that website use alone may not fully reflect the processes that contributed to weight loss in the parent trial [21].

### Strengths and Limitations

This study had several notable strengths. It examined website use among cancer survivors and their partners, an area with limited prior research, as few lifestyle interventions have studied website use among dyads. Our analysis also provided detailed page view analytics across the entire DUET website, offering insight into which website features were most frequently used. Furthermore, the study used validated measures to assess both diet quality and MVPA, enhancing the accuracy of the findings. However, like all studies, there were limitations. Most importantly, website data were only available for the 56 participants in the intervention arm, resulting in a relatively small analytic sample. A post hoc power calculation indicated that with this sample size, the study was powered (≥80%) only to detect correlations of approximately *r*≥0.40, representing a moderate to large effect size. As a result, smaller associations, particularly for accelerometer-measured MVPA and weight change, may not have been detectable, and the null findings for these outcomes should be interpreted with caution. Future web-based trials should consider recruiting larger samples to ensure adequate power to detect smaller associations between website use and clinically relevant outcomes such as weight change. Additionally, accelerometer-measured MVPA had 20% data missingness. As a result, complete-case analysis may introduce bias if participants with missing MVPA data differed meaningfully from those with complete data. Importantly, because multiple correlations were examined, there is an increased risk of type I error, and some associations may reflect spurious findings; therefore, the results should be interpreted within the exploratory, hypothesis-generating context of this secondary analysis. Similarly, it was not possible to determine whether participants viewed the full content of the weekly sessions they accessed, as data only represented whether participants accessed the sessions. Thus, it is likely that participants who briefly clicked into a session could therefore be coded similarly to those who reviewed the entire content, and time-stamp data may overestimate engagement if browser windows were left open. To partially address this limitation, we examined multiple measures of website use rather than relying on a single metric. Nevertheless, this measurement constraint may have led to imprecise estimates of website use and may help explain the modest strength of associations observed. Additionally, given the dyadic nature of the study, there may have been instances where dyads shared a single account and viewed website content together (as was documented in at least 1 case), potentially accounting for the difference between survivor and partner usage and underestimating individual-level usability. Another limitation is the demographic homogeneity of the sample, which primarily included female, non-Hispanic White breast cancer survivors residing in urban areas with higher socioeconomic status. As a result, these findings may not generalize to male survivors, racial and ethnic minority groups, individuals with lower income levels, or those living in rural or medically underserved settings.

### Conclusions

Minimal-touch lifestyle interventions delivered through web-based platforms are being implemented to promote diet and physical activity behaviors for weight management among cancer survivors. However, patterns of website use are often understudied. Findings from our study suggest that cancer survivors and their partners (especially older adults and females) actively used the DUET website, particularly the interactive e-learning sessions, and higher levels of website use were significantly associated with improvements in diet quality and self-reported MVPA. However, no statistically significant improvements were detected for accelerometry-measured MVPA or weight. These results highlight that web-based platforms may serve as a promising, scalable approach for delivering diet and physical activity guidelines and promoting healthy behaviors for long-term older cancer survivors and their partners. However, larger, more diverse dyadic web-based lifestyle interventions, including male survivors, racial and ethnic minority populations, individuals with lower income, and survivors in rural or underserved areas, are needed to confirm and expand upon these findings. Importantly, future web-based lifestyle interventions should also use more detailed engagement tracking (ie, content completion indicators, differentiation between brief access and full interaction with embedded activities, and minimum and maximum time thresholds) to distinguish brief access from full content consumption to strengthen the validity of engagement measures.

## Appendix

Multimedia Appendix 1 Distribution of DUET website page view activity for specific tools accessed by cancer survivors and their supportive partners, based on 967 total page views.

Multimedia Appendix 2 Distribution of DUET website page view activity for healthy eating, healthy weight, and exercise features accessed by cancer survivors and their chosen partners, based on 644 total page views.

## Abbreviations

ACS: American Cancer Society
CSIRO: Commonwealth Scientific and Industrial Research Organisation
DUET: Daughters, Dudes, Mothers, and Others Together
MVPA: moderate to vigorous physical activity
V&F: vegetable and fruit
WCRF/AICR: World Cancer Research Fund/American Institute for Cancer Research
WLM: Weight Loss Maintenance

## Data Availability

The data reported in this study are available on request from the corresponding author.

## References

[1] Bluethmann SM, Mariotto AB, Rowland JH (2016). Anticipating the "Silver Tsunami": prevalence trajectories and comorbidity burden among older cancer survivors in the United States. Cancer Epidemiol Biomarkers Prev.

[2] Wagle NS, Nogueira L, Devasia TP, Mariotto AB, Yabroff KR, Islami F, Jemal A, Alteri R, Ganz PA, Siegel RL (2025). Cancer treatment and survivorship statistics, 2025. CA Cancer J Clin.

[3] Shahrokni A, Wu AJ, Carter J, Lichtman SM (2016). Long-term toxicity of cancer treatment in older patients. Clin Geriatr Med.

[4] Heo J, Chun M, Oh Y, Noh OK, Kim L (2020). Metabolic comorbidities and medical institution utilization among breast cancer survivors: a national population-based study. Korean J Intern Med.

[5] Rock CL, Thomson CA, Sullivan KR, Howe CL, Kushi LH, Caan BJ, Neuhouser ML, Bandera EV, Wang Y, Robien K, Basen-Engquist KM, Brown JC, Courneya KS, Crane TE, Garcia DO, Grant BL, Hamilton KK, Hartman SJ, Kenfield SA, Martinez ME, Meyerhardt JA, Nekhlyudov L, Overholser L, Patel AV, Pinto BM, Platek ME, Rees-Punia E, Spees CK, Gapstur SM, McCullough ML (2022). American Cancer Society nutrition and physical activity guideline for cancer survivors. CA Cancer J Clin.

[6] Clinton SK, Giovannucci EL, Hursting SD (2020). The world cancer research fund/american institute for cancer research third expert report on diet, nutrition, physical activity, and cancer: impact and future directions. J Nutr.

[7] Baughman C, Norman K, Mukamal K (2024). Adherence to american cancer society nutrition and physical activity guidelines among cancer survivors. JAMA Oncol.

[8] Kaur H, Pisu M, Pekmezi DW, Rogers LQ, Martin MY, Fontaine KR, Waugaman KJ, Demark-Wahnefried W (2025). How healthy are the diets of cancer survivors? Characteristics of those most at risk and opportunities for improvement. J Natl Compr Canc Netw.

[9] Demark-Wahnefried W, Aziz NM, Rowland JH, Pinto BM (2005). Riding the crest of the teachable moment: promoting long-term health after the diagnosis of cancer. J Clin Oncol.

[10] Xu J, Hoover RL, Woodard N, Leeman J, Hirschey R (2023). A systematic review of dietary interventions for cancer survivors and their families or caregivers. Nutrients Dec.

[11] Sremanakova J, Sowerbutts AM, Todd C, Cooke R, Burden S (2021). Systematic review of behaviour change theories implementation in dietary interventions for people who have survived cancer. Nutrients.

[12] Jung Y, Chung J, Son H (2021). Physical activity interventions for colorectal cancer survivors: a systematic review and meta-analysis of randomized controlled trials. Cancer Nurs.

[13] Ficarra S, Thomas E, Bianco A, Gentile A, Thaller P, Grassadonio F, Papakonstantinou S, Schulz T, Olson N, Martin A, Wagner C, Nordström A, Hofmann H (2022). Impact of exercise interventions on physical fitness in breast cancer patients and survivors: a systematic review. Breast Cancer.

[14] Williams V, Brown N, Becks A, Pekmezi D, Demark-Wahnefried W (2020). Narrative review of web-based healthy lifestyle interventions for cancer survivors. Ann Rev Res.

[15] Lavoie A, Dubé V (2022). Web-based interventions to promote healthy lifestyles for older adults: scoping review. Interact J Med Res.

[16] Dee EC, Muralidhar V, Butler SS, Yu Z, Sha ST, Mahal BA, Nguyen PL, Sanford NN (2020). General and health-related internet use among cancer survivors in the United States: a 2013-2018 cross-sectional analysis. J Natl Compr Canc Netw.

[17] Holmes MM (2019). Why people living with and beyond cancer use the internet. Integr Cancer Ther.

[18] Williams VA, Brown NI, Johnson R, Ainsworth MC, Farrell D, Barnes M, Perumean-Chaney S, Fontaine K, Martin MY, Pekmezi D, Demark-Wahnefried W (2022). A Web-based lifestyle intervention for cancer survivors: feasibility and acceptability of survivorSHINE. J Cancer Educ.

[19] People Designs, Inc.. SURVIVORSHINE.

[20] Pekmezi D, Crane T, Oster R, Rogers L, Hoenemeyer T, Farrell D, Cole W, Wolin K, Badr H, Demark-Wahnefried W (2021). Rationale and methods for a randomized controlled trial of a dyadic, web-based, weight loss intervention among cancer survivors and Partners: the DUET study. Nutrients.

[21] Demark-Wahnefried W, Oster RA, Crane TE, Rogers LQ, Cole WW, Kaur H, Farrell D, Parrish KB, Badr HJ, Wolin KY, Pekmezi DW (2023). Results of DUET: a web-based weight loss randomized controlled feasibility trial among cancer survivors and their chosen partners. Cancers (Basel).

[22] Kaur H, Pavela G, Pekmezi DW, Rogers LQ, Cole WW, Parrish KB, Sayer RD, Wyatt HR, Demark-Wahnefried W (2023). Dietary barriers appear to influence the effects of a dyadic web-based lifestyle intervention on caloric intake and adiposity: a mediation analysis of the DUET trial. Nutrients.

[23] (1997). Health Behavior and Health Education: Theory, Research, and Practice. 2nd ed.

[24] Bandura A (1998). Health promotion from the perspective of social cognitive theory. Psychol Health.

[25] (2020). Adobe Acrobat Sign computer software. Version 6.0. Adobe Inc.

[26] People Designs, Inc. duet4health.

[27] Kelley HH (1997). The "stimulus field" for interpersonal phenomena: the source of language and thought about interpersonal events. Pers Soc Psychol Rev.

[28] Lewis MA, McBride CM, Pollak KI, Puleo E, Butterfield RM, Emmons KM (2006). Understanding health behavior change among couples: an interdependence and communal coping approach. Soc Sci Med.

[29] Demark-Wahnefried W, Jones LW, Snyder DC, Sloane RJ, Kimmick GG, Hughes DC, Badr HJ, Miller PE, Burke LE, Lipkus IM (2014). Daughters and Mothers Against Breast Cancer (DAMES): main outcomes of a randomized controlled trial of weight loss in overweight mothers with breast cancer and their overweight daughters. Cancer.

[30] Articulate G (2017). Articulate Storyline [computer software]. Version 360. Articulate Global, LLC.

[31] Frankenfield DC, Rowe WA, Smith J, Cooney R (2003). Validation of several established equations for resting metabolic rate in obese and nonobese people. J Am Diet Assoc.

[32] Fitbit Inspire 2 [wearable activity tracker]. Google LLC.

[33] Krebs-Smith SM, Pannucci TE, Subar AF, Kirkpatrick SI, Lerman JL, Tooze JA, Wilson MM, Reedy J (2018). Update of the healthy eating index: HEI-2015. J Acad Nutr Diet.

[34] (2025). Automated self-administered 24-hour dietary assessment tool. National Cancer Institute.

[35] Rogers LQ, McAuley E, Anton PM, Courneya KS, Vicari S, Hopkins-Price P, Verhulst S, Mocharnuk R, Hoelzer K (2012). Better exercise adherence after treatment for cancer (BEAT Cancer) study: rationale, design, and methods. Contemp Clin Trials.

[36] ActiGraph wGT3X-BT Activity Monitor. ActiGraph LLC.

[37] Amireault S, Godin G, Lacombe J, Sabiston CM (2015). Validation of the Godin-Shephard Leisure-Time Physical Activity Questionnaire classification coding system using accelerometer assessment among breast cancer survivors. J Cancer Surviv.

[38] Inc (2025). Zoom. Version 5.15.5. Zoom Video Communications, Inc.

[39] Hoenemeyer TW, Cole WW, Oster RA, Pekmezi DW, Pye A, Demark-Wahnefried W (2022). Test/retest reliability and validity of remote vs. in-person anthropometric and physical performance assessments in cancer survivors and supportive partners. Cancers (Basel).

[40] (2025). SAS Software, Version 9.4. SAS Institute Inc.

[41] Williams V, Brown N, Moore JX, Farrell D, Perumean-Chaney S, Schleicher E, Fontaine K, Demark-Wahnefried W, Pekmezi D (2022). Web-Based lifestyle interventions for survivors of cancer: usability study. JMIR Form Res.

[42] Paxton RJ, Hajek R, Newcomb P, Dobhal M, Borra S, Taylor WC, Parra-Medina D, Chang S, Courneya KS, Block G, Block T, Jones LA (2017). A lifestyle intervention via email in minority breast cancer survivors: randomized parallel-group feasibility study. JMIR Cancer.

[43] Van Blarigan EL, Kenfield S, Chan J, Van Loon K, Paciorek A, Zhang L, Chan H, Savoie M, Bocobo A, Liu V, Wong L, Laffan A, Atreya C, Miaskowski C, Fukuoka Y, Meyerhardt J, Venook A (2020). Feasibility and acceptability of a web-based dietary intervention with text messages for colorectal cancer: a randomized pilot trial. Cancer Epidemiol Biomarkers Prev.

[44] van den Berg SW, Peters EJ, Kraaijeveld JF, Gielissen MF, Prins JB (2013). Usage of a generic web-based self-management intervention for breast cancer survivors: substudy analysis of the BREATH trial. J Med Internet Res.

[45] Fredericks S, Martorella G, Catallo C (2015). A systematic review of web-based educational interventions. Clin Nurs Res.

[46] Chatterjee A, Prinz A, Gerdes M, Martinez S (2021). Digital interventions on healthy lifestyle management: systematic review. J Med Internet Res.

[47] Shi Y, Wakaba K, Kiyohara K, Hayashi F, Tsushita K, Nakata Y (2022). Effectiveness and components of web-based interventions on weight changes in adults who were overweight and obese: a systematic review with meta-analyses. Nutrients.

[48] Carr RM, Prestwich A, Kwasnicka D, Thøgersen-Ntoumani C, Gucciardi DF, Quested E, Hall LH, Ntoumanis N (2019). Dyadic interventions to promote physical activity and reduce sedentary behaviour: systematic review and meta-analysis. Health Psychol Rev.

[49] John JC, Ho J, Raber M, Basen-Engquist K, Jacobson L, Strong LL (2024). Dyad and group-based interventions in physical activity, diet, and weight loss: a systematic review of the evidence. J Behav Med.

[50] Graham SA, Stein N, Shemaj F, Branch OH, Paruthi J, Kanick SC (2021). Older adults engage with personalized digital coaching programs at rates that exceed those of younger adults. Front Digit Health.

[51] De Santis KK, Mergenthal L, Christianson L, Busskamp A, Vonstein C, Zeeb H (2023). Digital technologies for health promotion and disease prevention in older people: scoping review. J Med Internet Res.

[52] Bidmon S, Terlutter R (2015). Gender differences in searching for health information on the internet and the virtual patient-physician relationship in Germany: exploratory results on how Men and women differ and why. J Med Internet Res.

[53] Manierre MJ (2015). Gaps in knowledge: tracking and explaining gender differences in health information seeking. Soc Sci Med.

[54] Kebede AS, Ozolins L, Holst H, Galvin K (2022). Digital engagement of older adults: scoping review. J Med Internet Res.

[55] Pogrebnoy D, Dennett AM, Simpson DB, MacDonald-Wicks L, Patterson AJ, English C (2023). Effects of using websites on physical activity and diet quality for adults living with chronic health conditions: systematic review and meta-analysis. J Med Internet Res.

[56] Linke SE, Dunsiger SI, Gans KM, Hartman SJ, Pekmezi D, Larsen BA, Mendoza-Vasconez AS, Marcus BH (2019). Association between physical activity intervention website use and physical activity levels among Spanish-speaking Latinas: randomized controlled trial. J Med Internet Res.

[57] Hachiya J, Oliveira R (2024). The online community role in physical activity interventions. Procedia Computer Science.

[58] Funk KL, Stevens VJ, Appel LJ, Bauck A, Brantley PJ, Champagne CM, Coughlin J, Dalcin AT, Harvey-Berino J, Hollis JF, Jerome GJ, Kennedy BM, Lien LF, Myers VH, Samuel-Hodge C, Svetkey LP, Vollmer WM (2010). Associations of internet website use with weight change in a long-term weight loss maintenance program. J Med Internet Res.

[59] Hendrie GA, Baird DL, Brindal E, Williams G, Brand-Miller J, Muhlhausler B (2021). Weight loss and usage of an online commercial weight loss program (the CSIRO Total Wellbeing Diet Online) delivered in an everyday context: five-year evaluation in a community cohort. J Med Internet Res.

[60] Dent R, McPherson R, Harper M (2020). Factors affecting weight loss variability in obesity. Metabolism.

