# Supplementary figures and images for: Website Use and Associations With Behavior Change and Weight Loss in Cancer Survivors and Their Partners: Secondary Analysis of a Randomized Controlled Trial
Source: J Med Internet Res. 2026 Jan 30;28:e86908. doi: 10.2196/86908 (PMC12905566; doi:10.2196/86908)

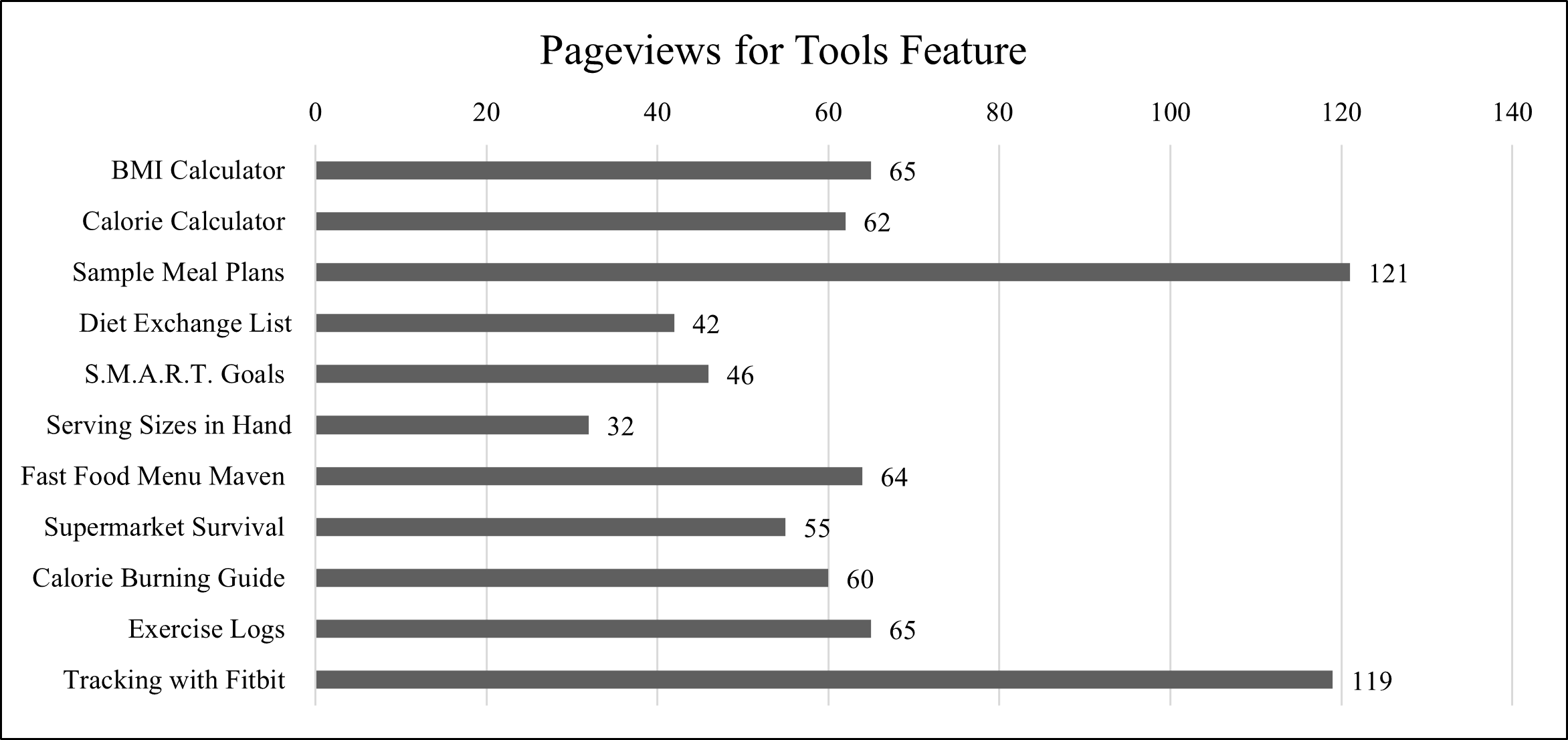

Supplement: Multimedia Appendix 1 [file jmir_v28i1e86908_app1.png]

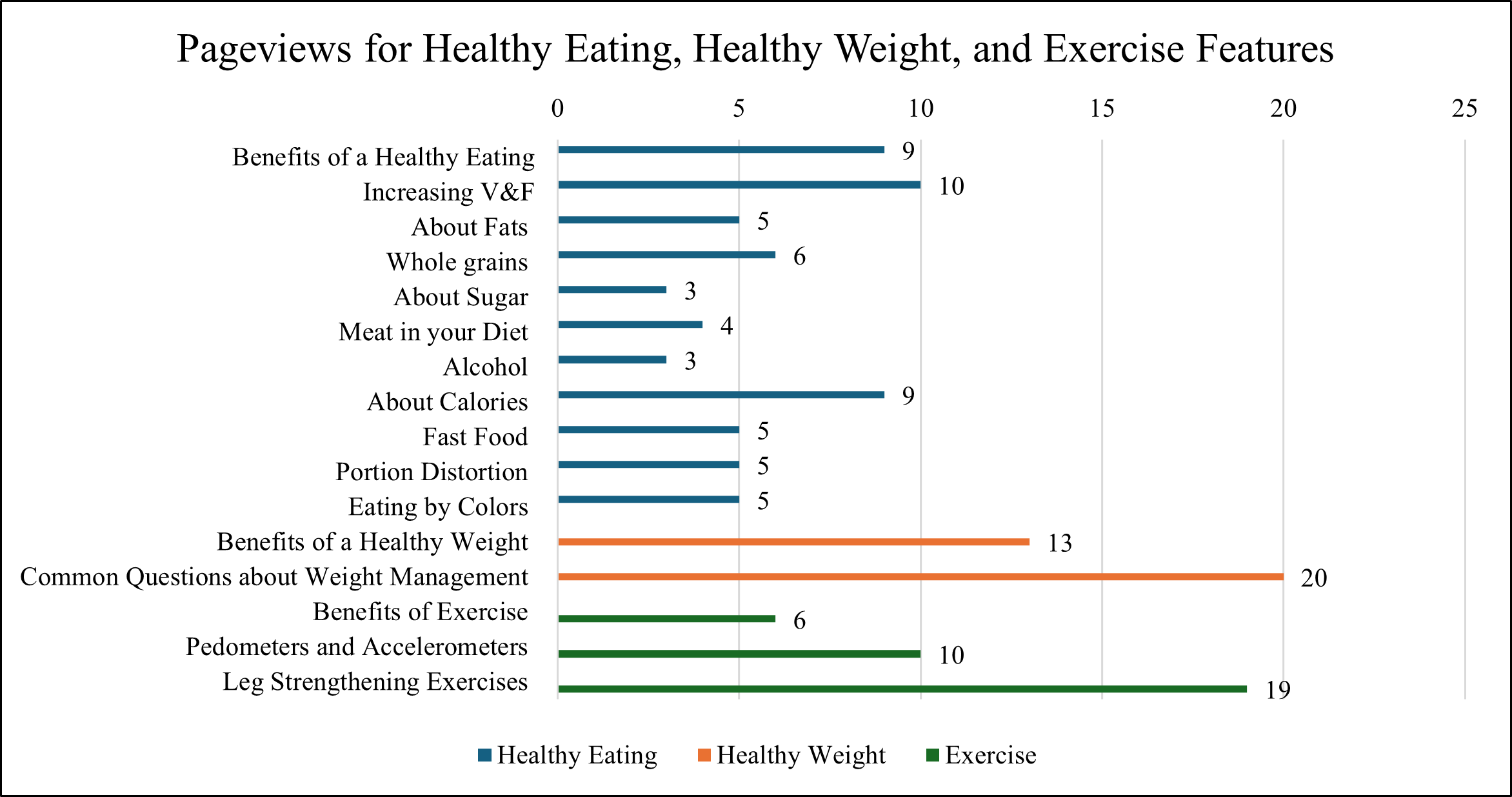

Supplement: Multimedia Appendix 2 [file jmir_v28i1e86908_app2.png]
